# Challenges of the public health response to a rare case of non-autochthonous *Mycobacterium leprae*, Ireland, 2024

**DOI:** 10.2807/1560-7917.ES.2025.30.3.2500033

**Published:** 2025-01-23

**Authors:** Rebecca Marshall, Eddie Horgan, Hugh Duane, Annette Dillon, Nadra Nurdin, Sarah O’Connell, Corinna Sadlier, Anne Dee, Anne Sheahan, Peter Barrett

**Affiliations:** 1 Department of Public Health, HSE South West, Cork, Ireland; 2 Department of Infectious Diseases, Cork University Hospital, Cork, Ireland; 3 Department of Medicine, University College Cork, Cork, Ireland; 4 Department of Public Health, HSE Mid-West, Limerick, Ireland; 5 Irish Centre for Maternal and Child Health Research, University College Cork, Cork, Ireland

**Keywords:** Leprosy, Public Health, Contact Tracing, Chemoprophylaxis, Neglected Tropical Disease, Stigma

## Abstract

This case report details the public health response to a multibacillary leprosy case in Ireland. The case presented with hypopigmented skin lesions and neurological symptoms. Challenges included delayed recognition in the clinical setting, contact tracing within a congregate setting and lack of specific Irish guidelines. Comprehensive contact tracing, chemoprophylaxis and follow-up care were implemented, guided by international protocols. This case underscores the need for tailored guidelines and stigma mitigation strategies for this neglected tropical disease in non-endemic regions.

Leprosy is one of 24 neglected tropical diseases (NTD) listed by the World Health Organisation (WHO) [1]. Although uncommon in Europe, leprosy contributes to a substantial burden of disease globally, with 182,815 new cases reported in 2023 [2]. Thirty-six countries in the European Region provided data on leprosy to the WHO in 2023, with 37 new cases of leprosy detected [2]. 

We present a case of non-autochthonous multibacillary (MB) leprosy with neurological involvement in summer 2024. This was one of five cases of leprosy notified in the Republic of Ireland in the last decade, and the first case in the south-west of the country in that time. It presented complex public health challenges, particularly with regard to contact tracing due to an absence of specific Irish or EU-level guidelines for the management of close contacts, as well as the stigmatising nature of this NTD.

## Case description

The case involved an individual in their 30s, living in a congregate setting in Ireland, who was born and grew up in a Caribbean country where leprosy remains endemic [4]. They moved to Ireland from southern Brazil 2 years before the diagnosis, after living in the region for 10 years, also an area with a high incidence of leprosy [5]. This individual initially presented to clinical services in late 2023 with a history of pain and numbness in the right arm and hand, and several raised hypopigmented lesions on the face, thorax, arms and legs. They underwent a number of investigations over 7 months before being diagnosed with *Mycobacterium leprae* on a skin biopsy in summer 2024 and received multi-drug therapy (MDT) with rifampicin, clofazimine, dapsone and steroids for neurological symptoms, which has resulted in recovery.

## Current public health guidance

In Ireland, there are no specific guidelines for the management of close contacts of *M. leprae* infection. We conducted a rapid scoping review of international guidelines and policy documents to inform our contact tracing strategy in real time, and our subsequent public health risk assessment.

While not generally considered to be a highly infectious disease, the exact mode of transmission of *M. leprae* is poorly understood. Although primarily transmitted through respiratory droplets, it generally requires prolonged periods of close contact for person-to-person transmission. Genetic factors appear to play a role in disease susceptibility, and it is estimated that 95–97% of the world’s population are naturally immune to the disease [6]. The incubation period is highly variable and typically ranges from 2 to 5 years but can be shorter or extend up to 20 years or more [7]. The attack rate varies by age, with younger individuals at a higher risk of acquiring and transmitting the infection upon exposure, and is also influenced by extreme poverty and poor nutrition [8].

Much of the existing international guidance relating to contact tracing comes from low-and middle-income countries, including settings where leprosy remains endemic [9,10]. Selective high-income countries have developed guidelines for the management of close contacts, including the United Kingdom (UK), United States (US) and Australia [7,11,12]. Nonetheless, there are notable inconsistencies in their approaches to infection prevention, contact tracing and post-exposure chemoprophylaxis.

## Contact tracing approach

There is no universally agreed definition of what constitutes a close contact for a case of leprosy. Some countries (e.g. UK and US) focus primarily on household contacts [11,12], while other jurisdictions provide a more generalised approach to contact tracing, including household and community contacts, e.g. India, Western Australia (WA) and the WHO [7,10,13].

We defined a close contact as any person who had been in contact with the untreated index case for at least 20 h per week, for at least 3 months in the preceding year, based on the WHO definition [10], and based on this undertook comprehensive contact tracing in household and occupational settings, and among social networks of the index case. 

Several international guidelines recommend contact tracing and examination of *all* household contacts, i.e. those living in the same dwelling or sharing the same kitchen or bathroom as the index case [7,10,11,13]. This guidance is likely to be based on the assumption that all household contacts form part of a nuclear family or, at a minimum, interact with one another and spend considerable periods of time together in shared living spaces. In countries where leprosy is endemic, it is likely that this recommendation also considers that over-crowding may be common, or housing quality may be suboptimal.

Our index case lived in a congregate setting with eight other adults. However, it became clear that they did not share living spaces with the majority of their housemates for any substantial periods of time, and we were unable to find tailored guidance to inform our public health risk assessment in this setting, which is increasingly common in Ireland. On detailed questioning, only one of the case’s housemates was considered to have interacted sufficiently with the case to be considered a close contact. In addition to this, two other individuals were identified as close contacts: the case’s partner and one work-related contact with whom they had spent prolonged periods of time. Of note, no credible source of infection was identified among their Irish contacts.

Ten other adults were considered potential close contacts, two of whom had since left the country (Figure). Given the highly stigmatising nature of leprosy infection [14,15] and its low infectivity [12], we considered that extensive contact tracing of all household members could be harmful to the index case, potentially jeopardising their housing or employment status, both of which were described as being informal or precarious.

**Figure:**
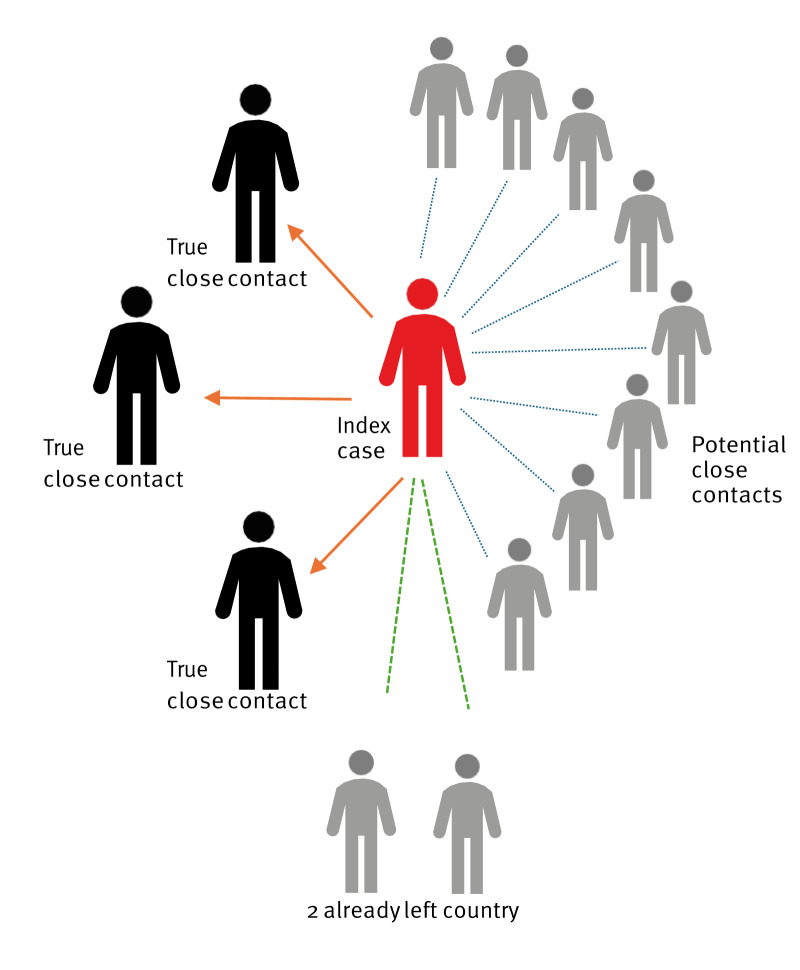
Contact tracing around a leprosy index case, Ireland, summer 2024 (n = 13)

## Chemoprophylaxis decisions

The WHO recommends chemoprophylaxis with single-dose rifampicin (SDR) for contacts of leprosy patients provided that active leprosy and tuberculosis (TB) have been excluded, and there are no other contraindications [9]. This treatment has been shown to have a protective effect in leprosy contacts when used in high incidence settings [16,17], and it is a well-tolerated, cost-effective, easy to administer preventative intervention for reducing disease transmission [17]. However, there is uncertainty regarding the applicability of these findings in areas of lower endemicity [18], which is reflected through the inconsistent approaches used in various international guidelines. In WA, chemoprophylaxis with SDR is offered to all household contacts of a newly diagnosed case of leprosy [7]. By contrast, SDR is not recommended in the US [11]. In the UK, chemoprophylaxis is also not routinely recommended, but it remains an option for contacts of a case with MB leprosy after public health risk assessment [12].

For our case of MB leprosy, we decided to offer chemoprophylaxis to the three true close contacts, based on the WHO, WA and UK guidelines.

## Follow-up care for close contacts

Due to the unusually long incubation period, most international guidelines, with the exception of those from the US [11], recommend annual follow-up for close contacts. Identifying cases early helps to prevent severe disabilities, prevents onward transmission of the disease, and reduces the social stigma tied to visible deformities [19]. However, the recommended duration of follow-up of contacts varies, typically ranging between 2 and 6 years [7,10,12].

For the three close contacts of our index case, arrangements were made with local primary care physicians to conduct yearly follow-up skin and neurological examinations for a minimum of 5 years, in accordance with WHO guidelines [10], and because all of the close contacts were migrants to Ireland from countries where leprosy remains endemic. Two of the three close contacts had not previously linked in with primary care services in Ireland and our regional Department of Public Health identified general practitioners willing to provide follow-up care.

## Discussion

Leprosy remains a burdensome NTD worldwide [2]. It is a chronic, curable granulomatous disease caused by infection with *Mycobacterium leprae.* The disease primarily affects the skin, peripheral nervous system, upper respiratory tract and eyes, and can lead to considerable disability if left untreated. While uncommonly reported in European countries, sporadic non-autochthonous cases remain a possibility, given high levels of international travel and shifting migration patterns [20]. Managing leprosy in non-endemic, high-income countries poses unique challenges. While it is a curable bacterial infection, diagnosis in low-prevalence settings is often delayed due to healthcare professionals' unfamiliarity with signs and symptoms of the disease, as observed in this case [21]. This delay increases the risk of irreversible nerve damage, which can lead to notable disability, requiring lifelong care and follow-up [9,22].

Furthermore, leprosy continues to be a highly stigmatised disease and those diagnosed require comprehensive psycho-social support that extends beyond medical treatment [22]. For migrants from endemic leprosy regions, the stigma surrounding the disease can be especially challenging. Many have precarious or informal employment and housing arrangements [23], which can heighten their reluctance to disclose contacts for fear of being singled out or further marginalised [14]. As such, when conducting contact tracing, it is crucial to handle the process with care to protect the individual’s privacy and to ensure their identity is not disclosed without their explicit consent [15].

Leprosy is one of 24 NTDs targeted for elimination under the WHO’s 2021–2030 Roadmap for NTDs. Achieving this goal will require support from all WHO member states, including those in non-endemic regions [24]. Most existing international guidelines for the management and prevention of the disease are tailored for countries with a high endemicity [9,10,13]. Where guidelines are available in high-income, non-endemic settings [7,11,12], there are notable variations in public health approaches to disease prevention and contact tracing, which adds to the complexities in managing this disease. 

## Conclusion

This report highlights the considerable public health challenges arising from the management of leprosy in a non-endemic, high-income setting. European countries should remain vigilant to the possibility of cases of leprosy arising and should consider developing tailored strategies to address this NTD within their specific contexts. These strategies should prioritise ongoing education for healthcare professionals to recognise and diagnose leprosy and other NTDs. They should also encompass robust measures to combat stigma and ensure access to comprehensive mental health support for affected cases. Moreover, we propose a more harmonised approach to the public health response to sporadic cases of leprosy across the European region. This should include greater standardisation across public health guidelines relating to contact tracing, post-exposure prophylaxis eligibility, and follow-up pathways for affected individuals and their contacts.
